# Health-related quality of life in racial and ethnic minority adults with type 2 diabetes: validity and responsiveness of the EQ-5D-3L

**DOI:** 10.1007/s11136-025-04070-2

**Published:** 2025-09-27

**Authors:** Mrinmayee Joshi, A. Simon Pickard, Kibum Kim, Lisa K. Sharp, Ben S. Gerber, Daniel R. Touchette

**Affiliations:** 1 Pharmacy Systems, Outcomes and Policy, University of Illinois Chicago College of Pharmacy, Chicago, IL USA; 2 Biobehavioral Nursing Science, University of Illinois Chicago College of Nursing, Chicago, IL USA; 3 Population and Quantitative Health Sciences, UMass Chan Medical School, Worcester, MA USA

**Keywords:** Type 2 diabetes, Health-related quality of life, EQ-5D, Minority health

## Abstract

**Purpose:**

Health-related quality of life (HRQL) assessment provides insights into the lived experiences of diverse populations. This study evaluated the convergent validity and responsiveness of the EQ-5D-3L in racial/ethnic minority populations with type 2 diabetes and elevated hemoglobin A1c (HbA1c).

**Methods:**

Secondary data from a clinical trial of a diabetes adherence support intervention for African-American and Latinx patients with type 2 diabetes (NCT02990299) were analyzed. Clinical (HbA1c, systolic blood pressure [SBP], body mass index [BMI]), psychological (brief 4-item diabetes distress scale [DDS4] for diabetes-related distress, 9-item patient health questionnaire [PHQ-9] for depressive symptoms), and HRQL (EQ-5D-3L) data were collected at baseline and every 6 months for 2 years. Convergent validity was assessed by examining the strength of associations between clinical/psychological measures and EQ-5D scores. A responder analysis using generalized estimating equation models was used to evaluate the EQ-5D's sensitivity to changes in clinical/psychological factors over time.

**Results:**

Among 221 individuals analyzed, HbA1c and SBP levels did not differ across those reporting varying levels of problems in any EQ-5D dimension. Individuals with higher BMI were more likely to report problems with mobility, usual activities, pain/discomfort, and anxiety/depression. DDS4 scores were moderately correlated with EQ-5D anxiety/depression dimension and index score, while PHQ-9 scores were strongly correlated with both. EQ-5D index score was insensitive to improvements in clinical measures but sensitive to improvements in psychological measures over time.

**Conclusion:**

The EQ-5D-3L captured variations in BMI and psychological measures but not in HbA1c and SBP. This study provides HRQL estimates that can be compared with other populations or studies.

**Supplementary Information:**

The online version contains supplementary material available at 10.1007/s11136-025-04070-2.

## Background

Type 2 diabetes is a chronic metabolic disorder characterized by hyperglycemia resulting from insulin resistance and impaired insulin secretion [1]. It is a leading public health concern, affecting about 37 million or 15% of the adult population in the United States, with prevalence rates continuing to rise [2]. Type 2 diabetes is a leading cause of morbidity and mortality, driven by complications such as cardiovascular disease and chronic kidney disease [3, 4]. The economic burden of type 2 diabetes is substantial, with costs arising from direct medical expenses, including medications, outpatient care, and hospitalizations, as well as indirect costs, such as lost productivity and informal caregiving [5].

Effective type 2 diabetes management requires a commitment to self-management behaviors such as blood glucose monitoring, adherence to medication plans, dietary adjustments, and engaging in consistent physical activity [6]. The constant need for disease management to prevent or delay complications can cause stress, frustration, and feelings of inadequacy [7]. These burdens can be particularly pronounced in individuals with multiple comorbidities, such as obesity or neuropathy, which can restrict mobility and interfere with usual activities [8]. Challenges with daily self-care including administering insulin or attending medical appointments are common, particularly in underserved populations with limited resources [9]. Many individuals experience diabetes-related distress characterized by emotional exhaustion and a sense of being overwhelmed by self-management demands, which is distinct from clinical depression [10]. Furthermore, clinical depression is also notably prevalent among individuals with type 2 diabetes, with research suggesting a bidirectional relationship where diabetes increases the risk of depression, and depression negatively influences diabetes management [11, 12].

Hyperglycemia in individuals with type 2 diabetes is associated with a greater comorbidity burden and physical symptoms like fatigue and neuropathic pain, which are closely linked to physical functioning and daily activities [13–15]. In addition to glycemic control, self-managing clinical conditions, such as blood pressure and body mass index (BMI), are essential aspects of care. Hypertension is common in those with type 2 diabetes and significantly increases the risk of cardiovascular disease, stroke, and kidney damage [16]. BMI is a crucial risk factor, with obesity shown to worsen insulin resistance and cause or exacerbate complications [17]. Obesity, which also often co-occurs with diabetes, has been linked to reduced physical activity, and self-stigma [18]. These factors collectively contribute to the overall burden of managing type 2 diabetes, particularly for individuals from underserved populations who face additional systemic barriers.

Systemic barriers to diabetes care not only complicate disease management but can also contribute to disparities in outcomes. Type 2 diabetes disproportionately impacts racial and ethnic minority populations, who frequently encounter limited access to care, financial constraints, and cultural and linguistic challenges [19, 20]. Managing the disease and fear of complications can be particularly overwhelming for those facing challenges in accessing medications, self-management resources, and regular healthcare check-ups [21]. Given the significant challenges faced by these historically underserved populations, it is critical to assess health-related quality of life (HRQL) to better understand and address their unique needs.

Accurate measurement of HRQL is essential for capturing the multidimensional impact of type 2 diabetes, guiding healthcare interventions, and informing policy decisions aimed at reducing disparities. The EQ-5D-3L is a widely used, preference-based instrument designed to measure HRQL across five key dimensions: mobility, self-care, usual activities, pain/discomfort, and anxiety/depression [22]. These dimensions are relevant in the context of type 2 diabetes, a condition associated with both physical limitations (e.g., fatigue, neuropathic pain, impaired mobility) and psychological challenges (e.g., emotional burden, diabetes distress, anxiety and depression). Unlike condition-specific instruments that focus narrowly on clinical or behavioral aspects, the EQ-5D-3L enables the simultaneous capture of both physical and mental health domains using a standardized format suitable for economic evaluation and population health surveillance. Despite the widespread use of EQ-5D, there is limited understanding of how well it captures the experiences of individuals from racial and ethnic minority groups living with type 2 diabetes. Clinical trials and population health studies often rely on standardized patient-reported outcome (PRO) instruments to capture health outcomes [23]. Therefore, examining the performance of these measures in different populations allows us to ensure their validity and enhance their applicability in diverse clinical and research settings. This study aimed to assess the validity and responsiveness of the EQ-5D-3L in racial and ethnic minority populations in the Chicago metropolitan region with type 2 diabetes and elevated HbA1c. A secondary objective was to estimate the prevalence of diabetes distress and depressive symptoms in the study population and to examine their relationships with clinical factors and overall HRQL.

## Methods

### Data source

This study was a secondary data analysis of clinical and PRO measure data collected in a randomized trial (NCT02990299). The trial evaluated the clinical impact of a clinical pharmacist and community health worker (CHW) team-based mobile health intervention for diabetes adherence support (mDAS). Detailed information on inclusion/exclusion criteria, data collection, participant characteristics, and main outcomes has been previously published [24, 25]. Study participants were recruited between March 2017 and January 2020, had type 2 diabetes with elevated HbA1c (≥ 8%), received care at the University of Illinois Hospital and Health Sciences System (UIH), and self-identified as belonging to African American or Black race, or Hispanic or Latinx ethnicity. Participants were randomized to either receive the mDAS intervention or usual care (waitlist control) for 1 year and crossed over to the alternate group for the subsequent year. Sociodemographic information for all participants was collected at baseline, and clinical factors and PROs were collected by interview at baseline, and every 6 months up to 2 years [24].

### Measures

#### EQ-5D-3L

The EQ-5D-3L is a preference-based measure of health status from the EuroQol group [22]. The questionnaire is comprised of a 5-item, 3-level descriptive system (EQ-5D-3L) asking respondents to indicate statements that best describe their health state on that day across 5 dimensions—mobility, self-care, usual activities, pain/discomfort, and anxiety/depression—using three response levels: no problems (1), some problems (2), and extreme problems (3). These dimensions represent a comprehensive yet parsimonious view of self-reported health status. Additionally, a visual analog scale (EQ VAS) asks respondents to rate their health on that day on a scale of 0 to 100, where 0 indicates worst health imaginable and 100 indicates best health imaginable. Responses for the EQ-5D-3L in each of the 5 dimensions were used to obtain an index-based utility score using the US value set, which ranges from -0.11 (extreme problems in all dimensions) to 1.00 (no problems in any dimension) based on societal preference weights [26]. The EQ-5D-3L has been widely used in health economic evaluations for patients with type 2 diabetes, with evidence supporting its validity, reliability and responsiveness in the population [27].

#### Clinical measures

For this study, glycemic control was measured using HbA1c, which indicates the average blood sugar level over the preceding 2–3 months [28]. HbA1c levels were assessed at baseline and follow-up visits and reported as percentages. Systolic blood pressure (SBP) was used to evaluate cardiovascular health, measured in millimeters of mercury (mmHg). SBP provides an important indicator of hypertension control, a common comorbidity in individuals with type 2 diabetes [16]. Body mass index (BMI), an indicator of adiposity, was calculated as weight in kilograms divided by the square of height in meters (kg/m^2^). These clinical measures served as key indicators of disease management.

#### Psychological measures

The brief diabetes distress scale (DDS4) was used to measure diabetes-related distress [29]. The 4-item questionnaire asked respondents to indicate the extent to which each of the following items bothered them over the past month: feeling overwhelmed with the demands of living with diabetes, often failing with their diabetes routine, not feeling motivated to keep up with diabetes self-management, and feeling angry, scared, and/or depressed when thinking about living with diabetes. Responses were on a 6-point Likert scale ranging from 1 to 2 for ‘Not a problem,’ 3–4 for ‘Moderate problem,’ and 5–6 for ‘A very serious problem.’ Mean item scores, ranging from 1 to 6, were obtained by summing up the scores for each item and dividing by the number of items [29]. Prior research suggests that clinically meaningful cut-points in terms of mean item score are: < 2.0 indicates ‘little or no distress’; ≥ 2.0–2.9 indicates ‘moderate distress’ and ≥ 3.0 indicates ‘high distress’. [30]

The patient health questionnaire (PHQ-9) was used to measure the severity of depressive symptoms [31]. The 9-item questionnaire asked patients to indicate how often they were bothered by symptoms commonly associated with depression, such as lack of interest or pleasure in activities, feelings of sadness, and difficulties with sleep or concentration, on a 4-item Likert scale from 0 for ‘Not at all’ to 3 for ‘Nearly every day’ over the past 2 weeks. Total scores, ranging from 0 to 27, were obtained by summing up the scores for each item. In terms of severity of depressive symptoms, total scores of 0–4 indicate minimal severity; 5–9, mild; 10–14, moderate; 15–19, moderately severe; and 20–27, severe [31].

### Statistical analysis

Participant characteristics were summarized using descriptive statistics, with continuous variables presented as means and standard deviations (SD) and categorical variables presented as counts and percentages. All statistical analyses were conducted using SAS 9.4 (SAS Institute, Cary, NC). Differences in HbA1c, SBP, and BMI levels by age, sex, race/ethnicity, education level, income level, insurance type, diabetes duration, and self-reported health status were assessed using Wilcoxon rank-sum tests for binary variables, Kruskal–Wallis tests for categorical variables with more than two levels, and Spearman correlation coefficients for continuous variables. Distributions of the EQ-5D-3L index score and EQ VAS score were assessed for normality using the Shapiro–Wilk test and non-parametric statistics were used for non-normal distributions. *p*-values < 0.05 were considered statistically significant for all statistical tests used in the study. Psychometric properties of the EQ-5D measured included convergent validity and responsiveness [32].

Responses to the EQ-5D, DDS4, and PHQ-9 were summarized for the overall cohort and by HbA1c, SBP, and BMI categories. The Kruskal–Wallis test was used to test for differences in HbA1c, SBP, and BMI levels in those reporting varying degrees of problems on the EQ-5D dimensions, and those with different DDS4 and PHQ-9 categories. Convergent validity was evaluated by assessing the strength of associations between the clinical and psychological measures, and EQ-5D dimension and summary scores. The strength of correlations, based on Spearman’s rank correlation coefficients, was classified as none (< 0.10), weak (0.10–0.29), moderate (0.30–0.49), and strong (> 0.49) [33]. We hypothesized that EQ-5D-3L index and VAS scores would be weakly correlated with asymptomatic clinical markers such as HbA1c and SBP, and moderately to strongly correlated with BMI and psychological measures. For descriptive and cross-sectional analyses, missing data were handled using pairwise deletion, which enabled the use of all available observations for each specific comparison.

The sensitivity of EQ-5D was assessed using a responder analysis approach, comparing participants who experienced improvements in clinical or psychological factors to those who did not. Generalized estimating equation (GEE) models were used to evaluate the association between improvements in clinical and psychological measures and EQ-5D scores over 6, 12, 18, and 24 months, relative to baseline. Improvements in predictor variables were coded as binary (yes/no) variables, defined as follows: HbA1c improvement: ≥ 0.5% reduction from baseline HbA1c [34], SBP improvement: follow-up SBP < 130 mm Hg (only in those with baseline SBP ≥ 130 mm Hg) [35], BMI improvement: ≥ 5% reduction compared to baseline BMI [36], diabetes distress improvement: > 0.25 reduction from baseline DDS4 mean item score [37], depressive symptoms improvement: ≥ 5 point reduction in PHQ-9 total score [38]. Each predictor was evaluated in a separate GEE model to assess its individual association with the HRQL outcomes, with adjustment for time and treatment assignment in the original study [24]. Models were constructed using an unstructured correlation structure, allowing the correlation between repeated measures within individuals to vary at different time points, and including all available data for each subject. We hypothesized that improvements in psychological measures would be associated with increases in EQ-5D-3L scores over time, while changes in clinical measures would have smaller or non-significant associations. Results were reported as parameter estimates, 95% confidence intervals (CI), and p-values.

Reporting of this study followed the COSMIN Reporting Guideline for studies on measurement properties of patient‑reported outcome measures: version 2.0 (Supplementary Materials) [39].

## Results

A total of 221 patients were included in the analysis. Their mean age was 55.2 years (SD 9.5). Approximately 70% self-identified as female, 67% as African American or Black, and 33% as Latinx or Hispanic (Table 1). More than 50% of the population reported a yearly household income below $20,000. The mean duration of diabetes in the sample was 12.7 years. Self-reported health status was distributed as follows: poor (11.3%), fair (47.5%), good (36.2%), very good (4.1%), and excellent (0.9%). Sociodemographic characteristics across HbA1c, SBP and BMI levels are provided in supplementary materials (Table S1). Individuals with higher HbA1c were more likely to be younger (*p* = 0.03). Individuals with higher SBP were more likely to be older (*p* < 0.01) and male (*p* < 0.01). Individuals with higher BMI were more likely to be African American/Black (*p* < 0.01) and have public insurance (*p* < 0.01).

**Table 1:** Baseline characteristics

Characteristic	Overall cohort (N = 221)
Age, years, mean (SD)	55.2 (9.5)
Sex, n (%)	
Female	154 (69.7)
Male	67 (30.3)
Race/ethnicity, n (%)	
African American	148 (67.0)
Latinx	73 (33.0)
Education, n (%)	
Less than high school	55 (25.0)
High school diploma or GED	55 (25.0)
Some college, 2-year certificate or associates degree	67 (30.5)
College graduate, some graduate school or graduate degree	43 (19.6)
Income, n (%)	
Less than $10,000	74 (34.1)
$10,000–$19,999	45 (20.7)
$20,000–$49,999	57 (26.3)
$50,000 or more	41 (18.9)
Insurance type, n (%)	
Public	139 (62.9)
Private	66 (29.9)
None/other	16 (7.2)
Duration of diabetes, years, mean (SD)	12.7 (7.8)
Self-reported health status, n (%)	
Poor	25 (11.3)
Fair	105 (47.5)
Good	80 (36.2)
Very good	9 (4.1)
Excellent	2 (0.9)
Clinical factors	
HbA1c, %, mean (SD)	9.2 (1.5)
SBP, mm Hg, mean (SD)	128.4 (16.3)
BMI, kg/m^2^, mean (SD)	35.6 (9.0)

*BMI* body mass index, *GED* general educational development, *HbA1c* glycated hemoglobin, *SBP* systolic blood pressure, *SD* standard deviation

More than 50% of individuals reported no problems with EQ-5D dimensions of self-care, usual activities, and anxiety/depression. 52.7% of individuals reported some problems with mobility and 51.1% reported some problems with pain/discomfort (Table 2). Overall, 40% of participants reported a ‘11,111’ health state, i.e., no problems with any dimensions. None reported extreme problems with mobility. The proportion of participants reporting extreme problems with other dimensions ranged from 0.5% for self-care to 22.2% for pain/discomfort. HbA1c and SBP levels did not significantly differ across individuals reporting varying levels of problems in any of the EQ-5D dimensions (*p* > 0.1 for all comparisons). Individuals with higher BMI were more likely to have more problems with mobility (*p* < 0.01), usual activities (*p* < 0.001), pain/discomfort (*p* < 0.01), and anxiety/depression (*p* = 0.01).

**Table 2:** Patient-reported outcomes for the overall cohort, and stratified by HbA1c, SBP, and BMI categories

	Overall cohort	HbA1c (%)				SBP (mm Hg)				BMI (kg/m^2^)			
	N = 221	≤ 8.0 (n = 48)	8.1–9.0 (n = 67)	9.1–10.0 (n = 49)	> 10.0 (n = 57)	< 120.0 (n = 62)	120–129.9 (n = 62)	130–139.9 (n = 53)	≥ 140.0 (n = 44)	< 30.0 (n = 69)	30.0–34.9 (n = 53)	35.0–39.9 (n = 46)	≥ 40.0 (n = 53)
EQ-MO, n(%)													
1	104 (47.3)	30 (62.5)	28 (41.8)	19 (38.8)	27 (47.4)	31 (50.0)	27 (43.6)	27 (50.9)	19 (43.2)	39 (56.5)	28 (52.8)	19 (41.3)	18 (34.0)
2	116 (52.7)	18 (37.5)	39 (58.2)	29 (59.2)	30 (52.6)	30 (48.4)	35 (56.5)	26 (49.1)	25 (56.8)	29 (42.0)	25 (47.2)	27 (58.7)	35 (66.0)
3	0 (0.0)	0 (0.0)	0 (0.0)	0 (0.0)	0 (0.0)	0 (0.0)	0 (0.0)	0 (0.0)	0 (0.0)	0 (0.0)	0 (0.0)	0 (0.0)	0 (0.0)
EQ-SC, n(%)													
1	183 (82.8)	42 (87.5)	53 (79.1)	43 (87.8)	45 (79.0)	54 (87.1)	50 (80.7)	41 (77.4)	38 (86.4)	58 (84.1)	44 (83.0)	41 (89.1)	40 (75.5)
2	37 (16.7)	6 (12.5)	13 (19.40)	6 (12.2)	12 (21.1)	8 (12.9)	12 (19.4)	11 (20.8)	6 (13.6)	11 (15.9)	9 (17.0)	4 (8.7)	13 (24.5)
3	1 (0.5)	0 (0.0)	1 (1.5)	0 (0.0)	0 (0.0)	0 (0.0)	0 (0.0)	1 (1.9)	0 (0.0)	0 (0.0)	0 (0.0)	1 (2.2)	0 (0.0)
EQ-UA, n(%)													
1	118 (53.4)	30 (62.5)	31 (46.3)	27 (55.1)	30 (52.6)	38 (61.3)	30 (48.4)	28 (52.8)	22 (50.0)	47 (68.1)	31 (58.5)	22 (47.8)	18 (34.0)
2	94 (42.5)	18 (37.5)	30 (44.8)	22 (44.9)	24 (42.1)	23 (37.1)	30 (48.4)	20 (37.7)	21 (47.7)	21 (30.4)	20 (37.7)	23 (50.0)	30 (56.6)
3	9 (4.07)	0 (0.0)	6 (9.0)	0 (0.0)	3 (5.3)	1 (1.6)	2 (3.2)	5 (9.4)	1 (2.3)	1 (1.5)	2 (3.8)	1 (2.2)	5 (9.4)
EQ-PD, n(%)													
1	59 (26.7)	17 (35.4)	16 (23.9)	11 (22.5)	15 (26.3)	21 (33.9)	15 (24.2)	12 (22.6)	11 (25.0)	24 (34.8)	13 (24.5)	10 (21.7)	12 (22.6)
2	113 (51.1)	24 (50.0)	31 (46.3)	27 (55.1)	31 (54.4)	26 (41.9)	33 (53.2)	29 (54.7)	25 (56.8)	34 (49.3)	32 (60.4)	27 (58.70)	20 (37.7)
3	49 (22.2)	7 (14.6)	20 (29.9)	11 (22.5)	11 (19.3)	15 (24.2)	14 (22.6)	12 (22.6)	8 (18.2)	11 (15.9)	8(15.1)	9 (19.6)	21 (39.6)
EQ-AD, n(%)													
1	134 (60.6)	34 (70.8)	41 (61.2)	27 (55.1)	32 (56.1)	40 (64.5)	34 (54.8)	32 (60.4)	28 (63.6)	51 (73.9)	30 (56.6)	29 (63.0)	24 (45.3)
2	76 (34.4)	13 (27.1)	24 (35.8)	16 (32.7)	23 (40.4)	17 (27.4)	25 (40.3)	20 (37.7)	14 (31.8)	17 (24.6)	19 (35.9)	16 (34.8)	24 (45.3)
3	11 (5.0)	1 (2.1)	2 (3.0)	6 (12.2)	2 (3.5)	5 (8.1)	3 (4.8)	1 (1.9)	2 (4.6)	1 (1.5)	4 (7.6)	1 (2.2)	5 (9.4)
EQ-5D index score, mean (SD)	0.65 (0.3)	0.67 (0.3)	0.63 (0.3)	0.64 (0.3)	0.66 (0.3)	0.73 (0.2)	0.61 (0.3)	0.63 (0.3)	0.65 (0.3)	0.73 (0.2)	0.67 (0.3)	0.65 (0.5)	0.53 (0.3)
EQ VAS, mean (SD)	61.9 (21.2)	66.5 (24.7)	71.5 (19.2)	64.7 (21.0)	67.5 (20.6)	72.5 (18.6)	64.9 (23.0)	66.4 (21.8)	67.3 (21.1)	71.5 (20.9)	66.5 (23.5)	68.9 (18.6)	63.6 (21.2)
DDS4^a^, n (%)													
Little or no distress	62 (28.1)	14 (29.2)	22 (32.8)	13 (26.5)	13 (22.8)	19 (30.7)	17 (27.4)	11 (20.8)	15 (34.1)	21 (30.4)	14 (26.4)	10 (21.7)	17 (32.1)
Moderate distress	50 (22.6)	14 (29.2)	13 (19.4)	12 (24.5)	11 (19.3)	19 (30.7)	12 (19.4)	9 (17.0)	10 (22.7)	20 (29.0)	12 (22.6)	7 (15.2)	11 (20.8)
High distress	109 (49.3)	20 (41.7)	32 (47.8)	24 (49.0)	33 (58.0)	24 (38.7)	33 (30.3)	33 (62.3)	19 (43.2)	28 (40.6)	27 (50.9)	29 (63.0)	25 (47.2)
PHQ-9^b^, n (%)													
Minimal	107 (48.4)	25 (53.2)	31 (46.3)	24 (49.0)	27 (47.4)	32 (51.6)	30 (49.2)	22 (41.5)	23 (52.3)	39 (56.5)	23 (44.2)	23 (50.0)	22 (41.5)
Mild	63 (28.5)	19 (40.4)	20 (29.9)	12 (24.5)	12 (21.1)	17 (27.4)	15 (24.6)	18 (34.0)	13 (29.6)	18 (26.1)	16 (30.8)	13 (28.3)	16 (30.2)
Moderate	30 (13.6)	2 (4.3)	13 (19.4)	8 (16.3)	7 (12.3)	8 (12.9)	10 (16.4)	7 (13.2)	5 (11.4)	8 (11.6)	8 (15.4)	6 (13.0)	8 (15.1)
Moderately severe	17 (7.7)	1 (2.1)	3 (4.5)	4 (8.2)	9 (15.8)	4 (6.5)	6 (9.8)	5 (9.4)	2 (4.6)	4 (5.8)	4 (7.7)	4 (8.7)	5 (9.4)
Severe	3 (0.0)	0 (0.0)	0 (0.0)	1 (2.0)	2 (3.5)	1 (1.6)	0 (0.0)	1 (1.9)	1 (2.3)	0 (0.0)	1 (1.9)	0 (0.0)	2 (3.8)

*BMI* body mass index (kg/m^2^), *DDS4* 4-item diabetes distress scale, *EQ-AD* EQ-5D anxiety/depression, *EQ-MO* EQ-5D mobility, *EQ-PD* EQ-5D pain/discomfort, *EQ-SC* EQ-5D self-care, *EQ-UA* EQ-5D usual activities, *EQ VAS* EQ-5D visual analog scale, *HbA1c* glycated hemoglobin (%), *PHQ-9* 9-item patient health questionnaire, *SBP* systolic blood pressure (mm Hg), *SD* standard deviation

^a^DDS4 categories are based on mean item score as follows: Little or no distress (< 2.0); moderate distress (≥ 2.0–2.9); high distress (≥ 3.0)

^b^PHQ-9 categories are based on total score as follows: Minimal severity of depressive symptoms (0–4); mild (5–9); moderate (10–14); moderately severe (15–19); severe (20–27)

The mean (SD) for the EQ-5D-3L index score in the overall cohort was 0.65 (0.27). Lower EQ-5D-3L index scores were associated with higher age (*p* < 0.01), female sex (*p* < 0.001), African American/Black race (*p* < 0.001), lower income (p < 0.001), having public insurance (*p* < 0.001), longer duration of diabetes (*p* = 0.01), lower self-reported health status (*p* < 0.01), and higher BMI (*p* < 0.001). Other sociodemographic characteristics were not significantly associated with EQ-5D-3L index scores.

The mean (SD) for the EQ VAS score in the overall cohort was 61.9 (21.2). Lower EQ VAS scores were associated with African American/Black race (*p* = 0.01), having public insurance (*p* < 0.01), and lower self-reported health status (*p* < 0.001). Other sociodemographic characteristics were not significantly associated with EQ VAS scores. An EQ VAS score of 0 was reported by 1 (0.5%) participant, and a score of 100 was reported by 16 (7.2%) participants.

Based on DDS4 mean item scores, 49.3% of the participants reported high diabetes distress (Table 2). The proportion of participants reporting high diabetes distress increased with increasing HbA1c, but the relationship between DDS4 categories and HbA1c was not statistically significant (*p* = 0.3). No relationship was observed between DDS4 categories and SBP or BMI. Based on PHQ-9 total scores, 48.4% of the participants reported minimal severity of depressive symptoms, 28.5% reported mild severity, and 22.6% reported moderate to severe depressive symptoms (Table 2). The proportion of participants reporting higher severity of depressive symptoms increased with increasing HbA1c levels (*p* < 0.01). No relationship was observed between PHQ-9 categories and SBP or BMI.

Examining the correlations between clinical measures, psychological measures, and EQ-5D scores showed that HbA1c and SBP were not correlated with any of the other measures (Table 3). Higher BMI was weakly correlated with higher PHQ-9 total score, higher EQ-5D-3L mobility, pain/discomfort, and anxiety/depression scores, and lower EQ VAS and EQ-5D-3L index scores, and moderately correlated with higher EQ-5D-3L usual activities score. A higher DDS4 score was weakly correlated with higher EQ-5D-3L mobility, self-care, usual activities, and pain/discomfort scores, moderately correlated with higher EQ-5D anxiety/depression scores, and lower EQ VAS and EQ-5D-3L index scores, and strongly correlated with higher PHQ-9 total scores. A higher PHQ-9 total score was moderately correlated with higher EQ-5D-3L mobility, self-care, pain/discomfort scores, and lower EQ VAS score, and strongly correlated with higher EQ-5D-3L usual activities and anxiety/depression scores, and lower EQ-5D-3L index score (Table 3).

**Table 3:** Correlations between clinical, psychological, and health-related quality of life measures

	HbA1c	SBP	BMI	DDS4	PHQ-9	EQ-MO	EQ-SC	EQ-UA	EQ-PD	EQ-AD	EQ VAS
HbA1c	1.00										
SBP	− 0.13	1.00									
BMI	0.00	0.04	1.00								
DDS4	0.11	− 0.0	0.08	1.00							
PHQ-9	0.10	0.06	0.14*	0.68****	1.00						
EQ-MO	0.10	0.03	0.22**	0.19**	0.47****	1.00					
EQ-SC	0.04	0.02	0.08	0.25***	0.35****	0.38****	1.00				
EQ-UA	0.07	0.08	0.31****	0.21**	0.50****	0.68****	0.50****	1.00			
EQ-PD	0.04	0.01	0.19**	0.23***	0.34****	0.55****	0.38****	0.50****	1.00		
EQ-AD	0.08	− 0.0	0.21**	0.41*	0.52****	0.34	0.17	0.36	0.32	1.00	
EQ VAS	− 0.03	− 0.08	− 0.17*	− 0.33****	− 0.40****	− 0.37****	− 0.29****	− 0.31****	− 0.35****	− 0.28****	1.00
EQ-5D-3L index score	− 0.09	− 0.02	− 0.28****	− 0.33****	− 0.56****	− 0.82****	− 0.56****	− 0.81****	− 0.83****	− 0.53****	0.44****

Strength of correlation based on Spearman’s rank correlation coefficient classified as none (< 0.10), weak (0.10–0.29), moderate (0.30–0.49), and strong (> 0.49); *p*-values * < 0.05, ** < 0.01, *** < 0.001, **** < 0.0001

*BMI* body mass index (kg/m^2^), *DDS4* 4-item diabetes distress scale (higher scores indicate more distress), *EQ-AD* EQ-5D anxiety/depression, *EQ-MO* EQ-5D mobility, *EQ-PD* EQ-5D pain/discomfort, *EQ-SC* EQ-5D self-care, *EQ-UA* EQ-5D usual activities, *EQ VAS* EQ-5D visual analog scale, *HbA1c* hemoglobin A1c (%), *SBP* systolic blood pressure (mm Hg), *PHQ-9* 9-item patient health questionnaire (higher scores indicate more severe depression)

The analysis of repeated measures of EQ-5D scores demonstrated their sensitivity to changes in clinical and psychological measures over time. The number of available observations declined over the 24-month follow-up due to attrition and intermittent missingness in clinical and PRO measures. Descriptive statistics for clinical and PRO measures, including the number of missing observations at each time point, are provided in supplementary materials (Table S2). Using GEE models, the EQ-5D-3L index score was not found to be sensitive to improvement in clinical measures over 24 months (Table 4). It measured a significant improvement in HRQL among individuals reporting a reduction in diabetes distress and depressive symptoms (Table 4). The EQ VAS was not sensitive to changes in clinical or psychological measures.

**Table 4:** Associations between improvements in clinical and psychological measures over 24 months, and EQ-5D scores: Results from generalized estimating equation models

Main predictor	EQ-5D-3L index score			EQ VAS score		
	Estimate (β)	95% CI	*p*-value	Estimate (β)	95% CI	*p*-value
HbA1c improvement	− 0.01	(− 0.03, 0.02)	0.63	− 0.03	(− 2.86, 2.81)	0.98
SBP improvement	0.00	(− 0.03, 0.03)	0.91	0.42	(− 3.09, 3.94)	0.81
BMI improvement	− 0.00	(− 0.04, 0.04)	0.85	− 1.58	(− 5.96, 2.79)	0.48
DDS4 improvement	0.03	(0.0, 0.06)	0.03	2.11	(− 0.98, 5.20)	0.18
PHQ-9 improvement	0.10	(0.06, 0.15)	< 0.001	1.03	(− 2.99, 5.05)	0.62

Each predictor was evaluated separately using a generalized estimating equation model with an unstructured correlation structure to account for within-individual correlations across repeated measures. Improvements in predictor variables are defined as follows: HbA1c: ≥ 0.5% reduction from baseline, SBP: follow-up SBP < 130 mm Hg (only in those with baseline SBP ≥ 130 mm Hg), BMI: ≥ 5% reduction compared to baseline, DDS4: > 0.25 reduction from baseline mean item score, PHQ-9: ≥ 5 point reduction in total score

*BMI* body mass index (kg/m^2^), *DDS4* 4-item diabetes distress scale (higher scores indicate more distress), *EQ VAS* EQ-5D visual analog scale, *HbA1c* hemoglobin A1c (%), *SBP* systolic blood pressure (mm Hg), *PHQ-9* 9-item patient health questionnaire (higher scores indicate more severe depression)

## Discussion

This study assessed the psychometric properties of the EQ-5D-3L in racial and ethnic minority populations with type 2 diabetes and elevated HbA1c receiving care at an academic health center in the Chicago metropolitan area. Results demonstrated that the EQ-5D-3L did not distinguish between groups with varying levels of HbA1c and SBP, nor did it capture changes in these clinical measures over time in the study population. Individuals with higher BMI reported more problems across multiple EQ-5D dimensions, including mobility, usual activities, pain/discomfort, and anxiety/depression, and had significantly lower EQ-5D-3L index scores. Nearly half of the participants reported a high level of diabetes distress, while about one-quarter reported moderate to severe depressive symptoms. Individuals with higher HbA1c reported a greater severity of depressive symptoms. Both diabetes distress and depressive symptoms were significantly associated with EQ-5D-3L index scores, supporting the instrument’s validity in capturing the psychological dimension of health.

The mean EQ-5D-3L index score in our study population (0.65) was lower than the US population-based estimate for diabetes (0.76) reported by Sullivan et al. [40]. This difference likely reflects disparities rooted in systemic inequities. The cohort analyzed in this study consisted predominantly of individuals from racial and ethnic minority groups with suboptimal control of type 2 diabetes (defined as HbA1c ≥ 8%) and a substantial proportion reporting low annual household incomes. Socioeconomic disadvantages and structural inequities faced by these groups negatively impact HRQL through greater comorbidity burden, barriers to accessing quality healthcare, lower health literacy, and higher financial and psychosocial stress compared to the general population [20]. Understanding and addressing systemic inequities is essential to improving HRQL and achieving health equity.

To contextualize our results within the broader body of evidence on psychometric properties of the EQ-5D-3L in diverse populations with type 2 diabetes, we compared our findings with those reported in the comprehensive literature review by Janssen et al. [27] There is mixed evidence in the literature on the ability of EQ-5D to discriminate between HbA1c levels. In our analysis, we did not observe an association between HbA1c levels and EQ-5D summary scores. This may be partially explained by the fact that our study population consisted entirely of individuals with elevated HbA1c, limiting the range of variability. Studies that reported a significant association between HbA1c levels and EQ-5D scores included participants across a broader spectrum of glycemic control [41, 42]. While elevated HbA1c is a strong risk factor for diabetes complications, its association with EQ-5D-3L scores may only become evident at extreme levels where symptoms and complications are more pronounced [43]. With regards to BMI, our findings align with prior research consistently demonstrating lower EQ-5D scores in groups with higher BMI [42, 44–47]. The ceiling effect observed in our study, with 40% of participants reporting perfect health, ‘11,111’ on the EQ-5D-3L, was consistent with some prior studies [48–50], suggesting limited sensitivity of the instrument in capturing subtle variations in HRQL. This can lead to the overlooking of less severe but important issues like mild pain or emotional distress, making it harder to compare different groups or evaluate the impact of treatments.

Our study found no correlation between EQ-5D-3L scoresand HbA1c or SBP—both largely asymptomatic clinical markers. BMI emerged as a clinical factor strongly associated with EQ-5D-3L scores. Over time, the EQ-5D-3L index score was found to be insensitive to clinical risk factor improvements but sensitive to patient-reported diabetes distress and depressive symptom improvement. Since the EQ-5D-3L index score reflects societal valuations rather than individual perceptions, it may not capture subtle improvements in clinical risk factors unless these translate to substantial functional or symptomatic changes. The EQ VAS was insensitive to improvements in clinical and psychological measures, as it is a direct self-rating and likely anchored to respondents’ overall well-being rather than specific clinical or psychological improvements.

The strong relationship between BMI and EQ-5D-3L scores suggests that interventions addressing weight could play a key role in enhancing HRQL outcomes. However, weight management is complex and influenced by various social and environmental factors. It is important to consider the broader context of patients’ lives when designing interventions to improve HRQL. Individuals in underserved communities may face challenges in their ability to engage in self-management, such as limited access to healthcare resources, healthy food, and safe environments for physical activity. Personalized approaches that focus on overall well-being and aim to reduce the psychological burden of disease management may better support patients in these settings [6]. Moreover, the demands of treatment, including regular glucose monitoring, medication regimens, and injections, can place emotional and/or financial strain on individuals, impacting their quality of life [51]. Continuous glucose monitoring may help to reduce the burden of frequent glucose checks and improve self-management [52–54]. Clear communication about the long-term benefits of managing clinical risk factors, combined with ongoing support, can also help patients better cope with the demands of living with type 2 diabetes, especially when systemic barriers are acknowledged and addressed [6].

The prevalence of diabetes distress and depressive symptoms in our cohort was consistent with that reported in a prior study describing the co-occurrence of these factors in a similar population [55]. While the prior study used the longer 17-item diabetes distress scale (DDS [56]), our study used the shorter DDS4 [29]. The DDS4 was developed as a streamlined version of DDS, incorporating a subset of items with the highest correlation to the DDS score. This brief instrument was designed with the intention of reducing respondent burden and enhancing its practicality in clinical and research settings [29]. Despite its brevity, the DDS4 captured a high prevalence of diabetes-related distress in our population, underscoring its potential utility in identifying individuals at risk of significant emotional burden related to diabetes management. Nonetheless, the shorter instrument may still be limited in its ability to capture the full breadth of emotional challenges. The tradeoff between comprehensiveness and practicality is an important consideration in selecting tools for assessing PROs. Future studies comparing the performance of the shorter DDS4 and DDS in various populations may help clarify the contexts in which each instrument is most appropriate.

The strong associations between diabetes distress, depressive symptoms, and overall HRQL in our study underscore the importance of mental health in diabetes care. These results align with prior research indicating that the emotional burden of diabetes management can profoundly impact HRQL [57]. Given the prevalence of diabetes distress and depression, interventions targeting emotional well-being as a central component of diabetes management should be prioritized. Culturally tailored approaches can be particularly helpful for racial and ethnic minority populations who may face additional challenges like stigma, limited access to mental health resources, and systemic inequities in healthcare. Furthermore, our study demonstrated that improvements in psychological factors are reflected in overall HRQL improvements over time. These findings highlight the need for holistic, patient-centered care models that address not only clinical control but also psychosocial aspects to achieve meaningful and sustained improvements in HRQL [58].

An important consideration for future research is the use of EQ-5D-5L, an updated version of the EQ-5D-3L that includes five levels of severity rather than three. The expanded response scale can offer improved responsiveness and better capture subtle variations in HRQL, especially in populations experiencing mild but meaningful changes [59, 60]. Prior studies have suggested that the EQ-5D-5L may reduce ceiling effects and enhance discrimination among patients with type 2 diabetes [61–63]. Future research comparing the measurement properties of the EQ-5D-3L and 5L in diverse diabetes populations would help determine the most appropriate instrument for use in clinical and research settings.

Our study addresses a critical gap by evaluating the psychometric properties of the EQ-5D-3L, specifically in a racial/ethnic minority population with type 2 diabetes and suboptimal glycemic control. It also contributes to the evidence on the psychological burden and overall HRQL in the population, shedding light on their relationships with key clinical factors. The findings emphasize the need for careful selection of PRO measures in clinical studies. Widely used measures like the EQ-5D-3L can offer valuable insights into overall HRQL. Its simplicity, ease of use and ability to generate a single summary index score make it a powerful tool. However, it may not fully capture specific challenges and nuances faced by individuals managing chronic conditions like type 2 diabetes. A notable strength of the study is the inclusion of repeated measures over a 2-year follow-up period, which allowed us to assess the longitudinal validity of the EQ-5D-3L. Additionally, the utility scores derived from the EQ-5D-3L can inform economic evaluation studies assessing the cost-effectiveness of interventions within this population.

However, there are limitations to consider. We could not assess the test–retest reliability of EQ-5D-3L as repeat assessments were conducted after several months, making it difficult to ensure stability in clinical and HRQL characteristics. The lack of data on comorbidities and complications limited our ability to fully account for factors that may mediate the relationship between clinical measures and HRQL. In the responder analysis, we adjusted for randomized group assignments but not treatment received at each time point in the original crossover study, where patients switched treatments after 12 months, which may have influenced the observed associations between changes in clinical or psychological measures and EQ-5D scores. As with many longitudinal studies, there was some attrition over the 24-month follow-up period, reflected in the declining number of observations across time points (Table S2). While missing data may introduce bias, we employed generalized estimating equation (GEE) models in our responder analyses, which are robust to unbalanced data and capable of incorporating all available observations [64]. Future studies may benefit from incorporating more extensive missing data handling approaches, such as multiple imputation, particularly in settings with substantial attrition. Moreover, the study was not explicitly powered to detect differences between participants who experienced clinical or psychological improvements versus those who did not. Consequently, some non-significant findings may be due to limited statistical power. Future research with larger samples may better clarify these relationships. Finally, since all participants in the study were recruited from a single urban academic health system and had elevated HbA1c at baseline, the findings may not be fully generalizable to individuals with better glycemic control, other racial and ethnic minority groups, or those receiving care in different geographic or clinical contexts. Although this study focused on minority populations which suggests limited generalizability, there is already evidence to support the strength and limitations of the EQ-5D-3L in broader general populations with diverse glycemic profiles or those receiving care in different healthcare settings [27].

## Conclusions

Consistent with other studies that have examined the properties of HRQL measures in type 2 diabetes, HbA1c and SBP were not associated with HRQL, while BMI and psychological measures showed significant correlations with EQ-5D scores. In addition to insights into the validity and responsiveness of the EQ-5D in historically underserved populations with suboptimal control of type 2 diabetes, our study results provide HRQL estimates that can be used for comparison with other populations or studies, contributing to a broader understanding of the health burden in diverse groups.

## Supplementary Information

Below is the link to the electronic supplementary material.

Supplementary Material 1

## Data Availability

The data that support the findings of this study are available from the corresponding author upon request.
