# Supplementary material for: Health-related quality of life in racial and ethnic minority adults with type 2 diabetes: validity and responsiveness of the EQ-5D-3L
Source: Qual Life Res. 2025 Sep 27;34(11):3147–58. doi: 10.1007/s11136-025-04070-2 (PMC12681497; doi:10.1007/s11136-025-04070-2)
Supplement: Supplementary file 1 — Supplementary Material 1 [file 11136_2025_4070_MOESM1_ESM.docx]

# Health-related quality of life in racial and ethnic minority adults with type 2 diabetes: Validity and responsiveness of the EQ-5D-3L

## Supplementary Materials

Table S1. HbA1c, SBP and BMI levels by baseline characteristics

|  | **Overall cohort** | **HbA1c (%)** | | | | | **SBP (mm Hg)** | | | | | **BMI (kg/m^2^)** | | | | |
| --- | --- | --- | --- | --- | --- | --- | --- | --- | --- | --- | --- | --- | --- | --- | --- | --- |
|  | N = 221 | ≤ 8.0  (n = 48) | 8.1 - 9.0  (n = 67) | 9.1 - 10.0  (n = 49) | >10.0  (n = 57) | p-value | <120.0  (n = 62) | 120 – 129.9  (n = 62) | 130 – 139.9  (n = 53) | ≥140.0  (n = 44) | p-value | <30.0  (n = 69) | 30.0 – 34.9 (n = 53) | 35.0 – 39.9 (n = 46) | ≥40.0  (n = 53) | p-value |
| **Age, years,**  **mean (SD)** | 55.2 (9.5) | 55.5 (11.7) | 56.7 (9.5) | 54.84 (8.1) | 53.60 (8.5) | 0.03^S^ | 53.0 (9.2) | 55.0 (10.2) | 55.1 (8.4) | 58.8 (9.4) | <0.01^S^ | 56.7 (8.6) | 55.1 (9.8) | 54.3 (9.3) | 54.2 (10.4) | 0.24^S^ |
| **Sex, n (%)** |  |  |  |  |  | 0.83^W^ |  |  |  |  | <0.001^W^ |  |  |  |  | 0.17^W^ |
| Female | 154 (69.7) | 37 (77.1) | 46 (68.7) | 31 (63.3) | 40 (70.18) |  | 52 (83.9) | 44 (71.0) | 33 (62.3) | 25 (56.8) |  | 43 (62.3) | 39 (73.6) | 32 (69.6) | 40 (75.5) |  |
| Male | 67 (30.3) | 11 (22.9) | 21 (31.3) | 18 (36.7) | 17 (29.82) |  | 10 (16.1) | 18 (29.0) | 20 (37.7) | 19 (43.2) |  | 26 (37.7) | 14 (26.4) | 14 (30.4) | 13 (24.5) |  |
| **Race/ethnicity n (%)** |  |  |  |  |  | 0.14^W^ |  |  |  |  | 0.07^W^ |  |  |  |  | <0.01^W^ |
| African American | 148 (67.0) | 27 (56.3) | 48 (71.6) | 32 (65.3) | 41 (71.9) |  | 37 (59.7) | 42 (67.7) | 33 (62.3) | 36 (81.8) |  | 37 (53.6) | 35 (66.0) | 33 (71.7) | 43 (81.1) |  |
| Latinx | 73 (33.0) | 21 (43.8) | 19 (28.4) | 17 (34.7) | 16 (28.1) |  | 25 (40.3) | 20 (32.3) | 20 (37.7) | 8 (18.2) |  | 32 (46.4) | 18 (34.0) | 13 (28.3) | 10 (18.9) |  |
| **Education, n (%)** |  |  |  |  |  | 0.70^K^ |  |  |  |  | 0.34^K^ |  |  |  |  | 0.59^K^ |
| Less than high school | 55 (25.0) | 13 (27.1) | 21 (31.3) | 9 (18.4) | 12 (21.4) |  | 21 (33.9) | 14 (22.6) | 13 (24.5) | 7 (16.3) |  | 25 (36.8) | 9 (17.0) | 7 (15.2) | 14 (26.4) |  |
| High school diploma or GED | 55 (25.0) | 12 (25.0) | 11 (16.4) | 15 (30.6) | 17 (30.4) |  | 13 (21.0) | 17 (27.4) | 13 (24.5) | 12 (27.9) |  | 15 (22.1) | 14 (26.4) | 14 (30.4) | 12 (22.6) |  |
| Some college, 2-year certificate or Associates degree | 67 (30.5) | 16 (33.3) | 19 (28.4) | 17 (34.7) | 15 (26.8) |  | 16 (25.8) | 17 (27.4) | 21 (39.6) | 13 (30.2) |  | 20 (29.4) | 13 (24.5) | 16 (34.8) | 18 (34.0) |  |
| College graduate, some graduate school or graduate degree | 43 (19.6) | 7 (14.6) | 16 (23.9) | 8 (16.3) | 12 (21.4) |  | 12 (19.4) | 14 (22.6) | 6 (11.3) | 11 (25.6) |  | 8 (11.7) | 17 (32.1) | 9 (19.6) | 9 (17.0) |  |
| **Income, n (%)** |  |  |  |  |  | 0.74^K^ |  |  |  |  | 0.50^K^ |  |  |  |  | 0.56^K^ |
| Less than $10,000 | 74 (34.1) | 19 (41.3) | 22 (33.3) | 13 (26.5) | 20 (35.7) |  | 18 (29.5) | 27 (45.0) | 17 (32.7) | 12 (27.27) |  | 25 (36.2) | 15 (28.3) | 14 (31.1) | 20 (40.0) |  |
| $10,000 to $19,999 | 45 (20.7) | 6 (13.0) | 17 (25.8) | 11 (22.4) | 11 (19.6) |  | 18 (29.5) | 6 (10.0) | 10 (19.2) | 11 (25.00) |  | 18 (26.1) | 10 (18.9) | 7 (15.6) | 10 (20.0) |  |
| $20,000 to $49,999 | 57 (26.3) | 11 (23.9) | 15 (22.7) | 14 (28.6) | 17 (30.4) |  | 15 (24.6) | 16 (26.7) | 17 (32.7) | 9 (20.45) |  | 17 (24.6) | 16 (30.2) | 14 (31.1) | 10 (20.0) |  |
| $50,000 or more | 41 (18.9) | 10 (21.7) | 12 (18.2) | 11 (22.4) | 8 (14.3) |  | 10 (16.4) | 11 (18.3) | 8 (15.4) | 12 (27.27) |  | 9 (13.0) | 12 (22.6) | 10 (22.2) | 10 (20.0) |  |
| **Insurance, n (%)** |  |  |  |  |  | 0.31^K^ |  |  |  |  | 0.46^K^ |  |  |  |  | 0.01^K^ |
| Public | 139 (62.9) | 28 (58.3) | 41 (61.2) | 33 (67.4) | 37 (64.9) |  | 35 (56.4) | 41 (66.1) | 29 (54.7) | 34 (77.3) |  | 40 (58.0) | 31 (58.5) | 27 (58.7) | 41 (77.4) |  |
| Private | 66 (29.9) | 18 (37.5) | 20 (29.8) | 13 (26.5) | 15 (26.3) |  | 21 (33.9) | 17 (27.4) | 19 (35.8) | 9 (20.4) |  | 18 (26.1) | 21 (39.6) | 16 (34.8) | 11 (20.8) |  |
| None/Other | 16 (7.2) | 2 (4.2) | 6 (9.0) | 3 (6.1) | 5 (8.8) |  | 6 (9.7) | 4 (6.4) | 5 (9.4) | 1 (2.3) |  | 11 (15.9) | 1 (1.9) | 3 (6.5) | 1 (1.9) |  |
| **Duration of diabetes, years, mean (SD)** | 12.70 (7.8) | 12.08 (7.4) | 11.76 (8.0) | 16.47 (7.9) | 11.09 (6.9) | 0.64^S^ | 12.2 (7.8) | 12.1 (7.1) | 14.0 (8.7) | 12.7 (7.6) | 0.45^S^ | 13.4 (7.7) | 12.4 (7.5) | 12.0 (7.7) | 12.6 (8.4) | 0.62^S^ |
| **Self-reported health status, n (%)** |  |  |  |  |  | 0.49^K^ |  |  |  |  | 0.09^K^ |  |  |  |  | 0.12^K^ |
| Poor | 25 (11.3) | 6 (12.5) | 8 (11.9) | 4 (8.2) | 7 (12.3) |  | 4 (6.4) | 6 (9.7) | 9 (17.0) | 6 (13.6) |  | 4 (5.8) | 6 (11.3) | 6 (13.0) | 9 (17.0) |  |
| Fair | 105 (47.5) | 22 (45.8) | 29 (43.3) | 26 (53.1) | 28 (49.1) |  | 29 (46.8) | 35 (56.4) | 24 (45.3) | 17 (38.6) |  | 35 (50.7) | 20 (37.7) | 24 (52.2) | 26 (49.1) |  |
| Good | 80 (36.2) | 16 (33.3) | 29 (43.3) | 16 (32.6) | 19 (33.3) |  | 24 (38.7) | 20 (32.4) | 19 (35.8) | 17 (38.6) |  | 23 (33.3) | 25 (47.2) | 15 (32.6) | 17 (32.1) |  |
| Very good | 9 (4.1) | 4 (8.3) | 1 (1.5) | 3 (6.1) | 1 (1.8) |  | 5 (8.1) | 1 (1.6) | 1 (1.9) | 2 (4.6) |  | 6 (8.7) | 1 (1.9) | 1 (2.2) | 1 (1.9) |  |
| Excellent | 2 (0.9) | 0 | 0 | 0 | 2 (3.5) |  | 0 | 0 | 0 | 2 (4.6) |  | 1 (1.4) | 1 (1.9) | 0 | 0 |  |

Note: p-values associated with ^K^Kruskal-Wallis test, ^W^Wilcoxon rank-sum test; ^S^Spearman correlation coefficient

BMI: body mass index; GED: general educational development; HbA1c: glycated hemoglobin; SBP: systolic blood pressure; SD: standard deviation

Table S2. Descriptive statistics of clinical and patient-reported outcomes measures at each data collection time point

|  | **Time point** | **N** | **%** | **Mean (SD)** |
| --- | --- | --- | --- | --- |
| **HbA1c, %** | **0 months** | 221 | 100% | 9.2 (1.5) |
|  | **6 months** | 195 | 88% | 8.8 (1.7) |
|  | **12 months** | 183 | 83% | 8.8 (1.7) |
|  | **18 months** | 183 | 83% | 8.6 (1.8) |
|  | **24 months** | 170 | 77% | 8.5 (1.8) |
| **SBP, mm Hg** | **0 months** | 221 | 100% | 128.4 (16.3) |
|  | **6 months** | 185 | 84% | 130.2 (16.8) |
|  | **12 months** | 146 | 66% | 130.0 (17.7) |
|  | **18 months** | 132 | 60% | 132.4 (18.6) |
|  | **24 months** | 114 | 52% | 130.4 (18.0) |
| **BMI, kg/m^2^** | **0 months** | 213 | 96% | 35.6 (9.0) |
|  | **6 months** | 174 | 79% | 35.8 (8.9) |
|  | **12 months** | 141 | 64% | 35.7 (9.0) |
|  | **18 months** | 125 | 57% | 35.3 (8.6) |
|  | **24 months** | 108 | 49% | 34.9 (9.0) |
| **DDS4 mean item score^a^** | **0 months** | 220 | 100% | 3.0 (1.5) |
|  | **6 months** | 201 | 91% | 2.7 (1.4) |
|  | **12 months** | 195 | 88% | 2.7 (1.4) |
|  | **18 months** | 189 | 86% | 2.7 (1.4) |
|  | **24 months** | 180 | 81% | 2.5 (1.4) |
| **PHQ-9 total score^b^** | **0 months** | 220 | 100% | 5.9 (5.2) |
|  | **6 months** | 199 | 90% | 5.6 (5.4) |
|  | **12 months** | 193 | 87% | 5.1 (5.2) |
|  | **18 months** | 187 | 85% | 5.0 (5.0) |
|  | **24 months** | 174 | 79% | 4.7 (5.4) |
| **EQ-5D-3L index score** | **0 months** | 220 | 100% | 0.65 (0.27) |
|  | **6 months** | 200 | 90% | 0.67 (0.25) |
|  | **12 months** | 195 | 88% | 0.66 (0.27) |
|  | **18 months** | 185 | 84% | 0.67 (0.25) |
|  | **24 months** | 177 | 80% | 0.69 (0.26) |
| **EQ VAS** | **0 months** | 221 | 100% | 67.9 (21.2) |
|  | **6 months** | 203 | 92% | 71.0 (21.9) |
|  | **12 months** | 195 | 88% | 70.4 (21.3) |
|  | **18 months** | 189 | 86% | 70.7 (21.1) |
|  | **24 months** | 180 | 81% | 73.2 (22.6) |

BMI: body mass index (kg/m^2^); DDS4: 4-item diabetes distress scale; EQ-5D-3L: 3-level version of the EQ-5D; EQ VAS: EQ-5D visual analog scale; HbA1c: glycated hemoglobin (%); PHQ-9: 9-item patient health questionnaire; SBP: systolic blood pressure (mm Hg); SD: standard deviation

^a^DDS4 mean item scores can range from 1 to 6, with higher scores indicating higher distress; ^b^PHQ-9 total scores can range from 0 to 27, with higher scores indicating higher severity of depressive symptoms
